# Poisoning by Phytolacca

**Published:** 1887-02

**Authors:** J. N. Mendenhall

**Affiliations:** Plano, Texas


### POISONING BY PHYTOLACCA.

*J. N. Mendenhall, P. B., M. D., Plano, Texas.*

For Daniel's Texas Medical Journal.

ON the night of February 21st, '85, I was hastily summoned to visit Mr. and Mrs. W., I found them vomiting and purging violently. I learned, upon inquiry, that they had eaten nothing unusual for supper except *horse-radish*. Alarming symptoms were soon developed in Mr. W. who had partaken more freely of the article than Mrs. W. He had a cold, clammy perspiration, pulsation and respiration imperceptible, passing blood and shreds of mucus membrane from his bowels, was indifferent as to the result, and indeed, was in a state of collapse. A judicious use of stimulants and astringent enemata soon revived him, and in two or three hours both patients were out of danger. An examination disclosed the fact that it was not horse-radish they had eaten, but poke root, a violent acro-narcotic poison. These two vegetables, or herbs, so frequently grow side by side in gardens, it is well to remember how to distinguish them. Horse-radish has a root 6–12 inches long, of a sharp, pungent taste, and causes lachrymation when cut, while poke root has a much larger, fleshy root, and is of sweetish bitter taste. Horse-radish will occasion, in some instances, vesical irritation, while poke root will act as a severe acro-narcotic poison, and may produce death.

